# Ethnoracial inequality and insurance coverage among Latino young adults

**DOI:** 10.1016/j.socscimed.2016.08.039

**Published:** 2016-11

**Authors:** Veronica Terriquez, Tiffany D. Joseph

**Affiliations:** a University of California, Santa Cruz, USA; b SUNY, Stony Brook, USA

**Keywords:** Affordable Care Act (ACA), Health reform, Latinos, Health care, Immigration, Transitions to adulthood

## Abstract

Previous research has demonstrated that Latino young adults are uninsured at higher rates relative to other ethnoracial groups. Recent implementation of the 2010 Affordable Care Act (ACA) has increased access to health insurance for young adults, in part by maintaining health coverage through their parents until age 26. This paper examines patterns of Latino young adults' insurance coverage during early ACA implementation by addressing three questions: 1) To what extent do Latino young adults remain uninsured relative to their peers of other ethnoracial groups? 2) How do young adults' family socioeconomic background, immigrant characteristics, college enrollment, and employment status mediate their coverage? And, 3) do patterns of insurance coverage differ for employer-provided coverage versus other sources of coverage (including parents’ health insurance)? Using a 2011 representative sample of U.S.-born and 1.5-generation immigrant young adults in California, we find that Latinos are more likely than other ethnoracial groups to remain uninsured. While they are as likely as similar peers to obtain employer-provided health insurance, they are less likely to possess insurance through other sources (including their parents). This study contributes to our understanding of the limits of the ACA in reducing disparities in insurance coverage for Latinos by highlighting the importance of family socioeconomic background, immigrant characteristics, college enrollment, and employment in shaping coverage among this age group.

## Highlights

• We examine ethnoracial disparities in Latino young adults' health insurance coverage.
• Latino young adults exhibit low rates of insurance coverage during early ACA implementation.
• Family SES, four-year college enrollment, and employment status predict coverage.
• Legal status, language use, and immigrant generation predict insurance coverage.
• In spite of low overall coverage, Latinos access coverage from employers similar to other groups.

## 1 Introduction

The passage and subsequent implementation of the Affordable Care Act (ACA) in recent years has led many policymakers and researchers to speculate that the reform will substantially reduce the number of uninsured Americans, including young adults eligible to maintain health coverage through their parents until age 26 (Hall and Rosenbaum, 2012, Ku, 2010). Latino young adults, both U.S.- and foreign-born, are one group with the potential to benefit significantly from the reform because of their high rates of un(der)-insurance relative to other ethnoracial groups, such as Whites, African-Americans, and Asian-Americans (Ortega et al., 2015). Understanding patterns of insurance coverage among this group is important because Latinos—the majority of whom come from immigrant families—are now the nation's largest ethnoracial minority. Moreover, in California and a few other states, Latinos make up the plurality of the young adult population.

Using data from the California Young Adult Study, this paper examines patterns of insurance coverage among Latino young adults ages 18–26 in California during the early stages of ACA implementation. We focus on U.S.-born and 1.5-generation immigrants (those born abroad and raised in the U.S.). These U.S.-raised young adults differ tremendously from recently arrived young adult migrants who tend to speak primarily Spanish (Rumbaut, 2004) and disproportionately encounter barriers to U.S. health insurance coverage (Bustamante et al., 2012, Bustamante et al., 2014, Ortega et al., 2015). Our empirical investigation examines the extent to which U.S.-born and 1.5-generation Latino young adults continue to exhibit lower rates of coverage relative to other ethnoracial groups while also accounting for factors that predict variation in coverage among this group. Our analyses uniquely capture the effects of family socioeconomic background, as well as immigrant characteristics and institutional access, in mediating the likelihood of any insurance coverage. We also explore how patterns of coverage differ for employer-provided insurance versus coverage by other sources. Our investigation contributes to an understanding of imperfect ACA implementation (Joseph, 2016) and how ethnoracial background, immigrant assimilation processes, legal documentation status, postsecondary enrollment, and labor market participation shape disparities in young adults’ health coverage.

### 1.1 The Affordable Care Act and other sources of health insurance for young adults

The 2010 ACA heralded a historic effort that would transform the U.S. healthcare system by increasing access to insurance coverage, making health care more affordable, and creating more efficiency in the healthcare system (Hall and Rosenbaum, 2012, Ku, 2010). As initially written, the ACA was expected to insure nearly 32 million more Americans (Hall and Rosenbaum, 2012). Implemented incrementally beginning in September 2010, the reform aimed to ensure that every eligible individual obtained preventive care coverage. Among its various provisions, the ACA reform in 2013–14 expanded subsidized Medicaid coverage for low-income individuals, created federal and state marketplaces where private insurance companies could sell plans to middle and higher-income individuals ineligible for the Medicaid expansion, and imposed penalties on employers with 10 + employees who did not provide coverage for their employees (Levy, 2013, Patel and McDonough, 2010). But before these provisions were implemented, the ACA enabled young adults up to age 26 to enroll in or remain on their parents' insurance plans even if they were “married, not living with their parents, attending school, not financially dependent on their parents, or not eligible to enroll in their employer's plan” (DHHS, 2014). This ACA provision went into effect for plans renewing on or after September 23, 2010. In spite of political resistance to the policy and its implementation, an estimated 3 million young adults benefited from this provision by September 2011, with the largest gains seen among nonstudents, the unmarried, and men (Sommers et al., 2013). As Patel and McDonough (2010) have noted, significant efforts were also made to encourage remaining uninsured young adults to purchase coverage in the marketplace, as this age-group tends to be healthier and generate fewer healthcare costs. This age group has also tended to be uninsured due to inability to afford coverage and working in jobs that did not provide coverage. In theory, the premiums paid by this group were intended to subsidize the costs of older, sicker, and costlier patients in marketplace insurance plans. This meant that the success of the marketplace was tied to young adults' participation.

Despite gains in coverage under the ACA for young adults and the general population, Latinos of various ages were the least informed about ACA benefits and have had the lowest enrollment numbers compared to other ethnoracial groups (Carey, 2015, Mosqueira and Sommers, 2016). A similar trend has been found in California, where it is projected that nearly three-fourths of the state's remaining uninsured will be Latino by 2019 (Lucia et al., 2015).

Aside from this ACA provision, young adults also could have accessed insurance from other sources. Like nonelderly adults, they could purchase out-of-pocket individual private insurance. Given their age, many could potentially obtain coverage through four-year postsecondary institutions, which tend to offer comprehensive plans for this age group (Turner and Hurley, 2002). Attending and graduating from college typically also leads to job and earnings opportunities that increase access to insurance (Cantor et al., 2012). Yet it is well known that Latino young adults enroll in four-year colleges at low rates given their low family socioeconomic background, unequal access to quality K-12 education, disadvantages related to their parents’ or their own precarious documentation status, and persisting ethnoracial discrimination (Terriquez, 2014; Bean et al., 2013, Posselt et al., 2012). Four-year college institutions are therefore unlikely to function as an insurance access point for many Latino young adults.

However, despite their comparatively low enrollment rates in four-year postsecondary institutions, Latino young adults exhibit similar or higher labor market participation rates than other ethnoracial groups (Terriquez, 2014). Neither an immigrant background nor a disadvantaged socioeconomic position necessarily impedes Latinos’ labor market participation (Waldinger et al., 2007), especially among young men (Bachmeier and Bean, 2011). As such, employers may serve as a key source of coverage for Latino young adults, possibly helping to equalize their access to insurance—especially for those raised in the U.S. who tend to find better paying jobs offering insurance benefits when compared to more recent arrivals (Waldinger et al., 2007). However, it is also possible that some Latino young adults end up in low-status jobs that do not provide insurance. Previous research finds that lack of employer-provided coverage generally contributes to low insurance rates for Latinos, as some work in occupations (i.e. food service, agriculture, personal care) that generally do not provide coverage (Buchmueller et al., 2007, Ortega et al., 2015). It therefore remains an open question whether employer-provided coverage ameliorates or exacerbates ethnoracial inequalities in health coverage for U.S.-raised Latinos. Further, given that Latino young adults do not remain disadvantaged in labor market participation relative to other ethnoracial groups, the determinants of employer-based insurance coverage merit exploration.

### 1.2 Socio-demographic correlates of health insurance coverage

Latinos can sometimes face significant barriers to accessing health insurance. Notably, the legacy of racism and ongoing racial discrimination contributes to lower healthcare access for all racial minority groups (Feagin and Bennefield, 2014, LaVeist and Isaac, 2012). However, Latinos tend to obtain insurance at lower rates than other racial minorities (Alegria et al., 2012; Ortega et al., 2015, Viruell-Fuentes et al., 2012). Furthermore, Latinos represent a wide range of ethnicities and have much phenotypical diversity given the history of “mestizaje” (race mixing) in Latin America. This means that Latinos’ experiences accessing healthcare differ depending on whether they are racialized by others as light/white, dark/black, or mestizo (LaVeist and Isaac, 2012). These phenotypical differences create structural advantages for lighter/white Latinos relative to mestizo and darker/black Latinos in the larger society, but also with regard to health outcomes and experiences in the healthcare system (LaVeist and Isaac, 2012).

Latino young adults also disproportionately come from lower socioeconomic backgrounds in terms of education and income. Consequently, they encounter challenges to purchasing private insurance or obtaining it through family members (Ortega et al., 2015). And due to racial and socioeconomic segregation, Latinos tend to live in neighborhoods with less access to affordable and quality healthcare (Hargraves and Hadley, 2003, Hadley, 2003), and this may deter individuals from obtaining insurance.

Another important structural barrier to coverage for some immigrant Latinos is documentation status, which has become increasingly important for accessing public goods (Fox, 2016, Light, 2012). It has been estimated that nearly 48 percent of the almost 17.6 million Latin Americans living in the U.S. are undocumented, with the majority being from Mexico and Central America (Wallace, 2015). Thus, immigrant young adults are especially affected by rigidly-enforced public policies that have become harsher towards noncitizens since the mid-1970s (Brown, 2013, Fox, 2016, Park, 2011). In particular, the 1996 welfare and immigration reforms, namely the Illegal Immigration Reform and Immigration Responsibility Act (IIRIRA) and Personal Responsibility and Work Opportunity Reconciliation Act (PRWORA), make significant legal distinctions between individuals residing in this country depending on whether they are U.S. citizens, long-term legal permanent residents (LPRs), visa holders, or undocumented (Marrow and Joseph, 2015, Portes et al., 2012).

A present-day consequence of the IIRIRA and PRWORA reforms, passed nearly 20 years ago, is most immigrants’ exclusion from ACA provisions. Only citizens and long-term immigrants with legal permanent residency or asylum are eligible. This means that legal permanent residents with fewer than five years in the country, along with unauthorized immigrants and student or work visa holders, are excluded from the Medicaid expansion and purchasing coverage in the exchanges (even with their own money) (Bustamante et al., 2012, Bustamante et al., 2014, Ortega et al., 2015). Consequently, most of these immigrants will remain uninsured unless they live in subnational jurisdictions that provide coverage eligibility using non-federal funds (Joseph, 2016, Marrow and Joseph, 2015, Zuckerman et al., 2011). This will also limit the health coverage of a minority of 1.5-generation Latino immigrants who lack legal documentation status (Castañeda and Melo, 2014). Moreover, in the rare instances where they can afford or obtain insurance from an employer or postsecondary institution, fears about disclosing their status may also prevent some undocumented young adults from signing up for insurance (Berk et al., 2000, Hacker et al., 2011, Ortega et al., 2015).

The legal status of immigrant parents may also play a role in shaping access to insurance. Some citizens and LPRs come from mixed-status families in which their immigrant parents remain undocumented and consequently do not have insurance (Castañeda and Melo, 2014, Ortega et al., 2015). These Latino young adults are unlikely to benefit from the ACA provision extending insurance through parental coverage.

While the previously discussed structural factors may shape Latino young adults’ healthcare access, immigrant assimilation theories potentially offer further insights into patterns of insurance coverage. Generally, immigrants and their descendants increasingly resemble the native-born population the longer they and their families live in the country (Alba and Nee, 2003). Yet ethnic groups vary in the extent to, and pace at, which they achieve socio-economic success (often measured by educational and occupational attainment) (Portes and Rumbaut, 2014). In some cases, the 1.5 and second generations outperform the native-born population (or the third-plus generations) by some indicators of success, and thus benefit from “second-generation advantage” (Kasinitz et al., 2008). This may be because the children of immigrants sometimes exhibit an “immigrant optimism” (Kao and Tienda, 1995) and resourcefulness that enable them to improve their situations, despite socioeconomic challenges and racial discrimination (Kasinitz et al., 2008). Therefore, we might expect first-generation migrants to have low rates of coverage but their young adult children—the 1.5 and second generation—to have higher rates relative to peers from non-immigrant families.

Alternatively, we might expect young adults from immigrant families to be disproportionately uninsured given that immigrants and their offspring tend to display overall low rates of health insurance coverage (Huang et al., 2006). While socioeconomic and structural barriers drive these low coverage rates, some aspects of culture may also play a role. While we recognize that Latino culture is poorly conceptualized in health research (Hunt et al., 2004), it is worth noting that Latino immigrants and their children exhibit healthier behaviors (by some measures) and report better health than the native-born population from similar socioeconomic backgrounds (Acevedo-Garcia and Bates, 2008, Alegria et al., 2012). Thus, comparatively better health may reduce the perceived need for health insurance. Furthermore, some Latinos may turn to service providers that practice traditional medicine such as *curanderos* (general-purpose healers), *sobadores* (massage therapists), and *parteras* (midwives), who may not take insurance (Portes et al., 2012). Latinos living close to the southern border or who can take a short plane ride to the Caribbean may also seek culturally familiar and relatively inexpensive care outside of the country (Portes et al., 2012, Ortega et al., 2015), thus decreasing reliance on U.S. insurance.

Indeed, research has yet to adequately measure aspects of culture that may impact health-related behaviors (Hunt et al., 2004). Absent of such measures, the ability to speak parents' native tongue represents a rough indicator of ethnic cultural ties for the U.S. raised children of immigrants (Portes and Rumbaut, 2001). Consequently, it may be possible that some U.S.-raised Latinos who grew up speaking Spanish (or another language spoken by their parents) to engage in cultural practices or display cultural preferences that lead them to opt out of obtaining health insurance. Because U.S.-raised Latinos typically speak English (Alba et al., 2002, Rumbaut, 2004), those who also speak their parents' native language do not necessarily encounter language barriers to accessing insurance, as might their parents or other first-generation immigrants.

### 1.3 Present study

This investigation focuses on young adults in California, where a quarter of the Latinos in the country reside. We examine patterns of Latino young adults' insurance coverage during early ACA implementation for 18–26-year-olds who are eligible for coverage under their parents’ insurance. (Our study excludes individuals who migrated as young adults). We address three interrelated research questions:

1) To what extent do Latino young adults remain disproportionately uninsured compared with their peers from other ethnoracial backgrounds?
2) How do Latino young adults' family socioeconomic background, immigrant characteristics, college enrollment, and employment status mediate their likelihood of health insurance coverage?
3) Do patterns of insurance coverage differ for employer-provided insurance versus coverage by other sources of insurance (including parents)?

Understanding Latino young adults' coverage in California is important not only because of the size of the population, but also because trends in the state may provide insights into patterns of young adults' coverage around the country. It is worth noting that in California, over three quarters of Latinos claim Mexican or Central American ancestry, similar to 71% of Latinos nationwide (Ennis et al., 2010). Although Mexicans and Central Americans have different histories of migration, they encounter similar contexts of reception and treatment by out-group members (Davenport et al., 2002, Portes and Rumbaut, 2014). These two groups disproportionately encounter challenges posed by their own or family members' precarious legal statuses (Ortega et al., 2015). Thus, Latino young adults' coverage as examined in this study largely reflect the experiences of individuals of Mexican and Central American descent. Latinos of South American and Caribbean origin are not well-represented in this study.

## 2 Methods

### 2.1 Data

Our analysis relies on telephone survey data from the California Young Adult Study, a mixed-methods investigation of transitions to adulthood among 18–26-year-olds. Survey data come from telephone and cell phone interviews conducted in 2011 with 2200 randomly selected youth who attended school in California before the age of 17. The survey sample excludes first generation young adult migrants and international students. Young adults residing in high poverty census tracts were oversampled. The sample contains 1021 Latinos who self-classified as Latino, Hispanic, Chicano, or other Latin American, regardless of other racial identification. This study sample also includes 772 Non-Hispanic Whites, 212 Non-Hispanic Asian-Pacific Islanders, 119 Non-Hispanic Blacks, and 76 individuals who self-classified as some other race. When sampling weights are used in data analysis, results reflect the ethnoracial, Latino immigrant, and income background of 18–26-year-olds in California who were born in the U.S. or immigrated as minors. Unlike larger data sets such as the American Community Survey, Current Population Survey, and California Health Interview Survey, these data uniquely contain measures of legal status, socioeconomic status of respondents' families of origin, and type of college attended—all potential predictors of Latino young adults’ health coverage.

### 2.2 Measures and analytic strategy

We begin our analysis by providing separate descriptive statistics for the entire sample and for Latinos. To address our first research question, we use logistic regressions to examine ethnoracial differences in whether or not respondents had any insurance. For our second question, we account for various factors that might explain ethnoracial inequalities in coverage, and examine how these factors influence coverage among Latinos. Finally, we use multinomial logistic regressions to address our third question regarding patterns of employer-provided insurance versus coverage by other forms of insurance (including through parents). Our categorical dependent variable—respondent's type of insurance coverage—contains three categories: 1) no health insurance, 2) employer-provided health insurance, and 3) health insurance provided through their parents (perhaps due to the ACA) or some other source. Unfortunately, our data do not allow us to determine how respondents obtained their insurance, with the exception of employer-provided insurance.

In the first set of models, which examine if respondents have insurance (and what type), we assess ethnoracial differences in coverage, after controlling for gender and age. We use Latinos as the reference category because they comprise a plurality of the sample, and we are interested in how they compare to other ethnoracial groups.

In the second set of models, we account for family socioeconomic status using measures of parental education and family income. To operationalize parental education, we identify youth who were raised by parents who did not possess a high school degree or equivalent and those who had at least one parent with a bachelor's degree or more. We use young adults who have at least one parent with a high school degree or some college but no bachelor's degree as the reference group. We also identify those who come from a low-income background, defined by free and reduced lunch eligibility in high school or parental reliance on public assistance while respondents were teens.

Next, in a third set of models we assess the role of immigrant background characteristics in mediating young adults' likelihood of being insured, in addition to variables from the previous models. Similar to other studies of children of immigrants (Kasinitz et al., 2008, Portes and Rumbaut, 2001), we identify 1.5 and second generation youth as those with at least one foreign-born parent as coming from an immigrant family. Those from non-immigrant families have U.S.-born parents and are third generation-plus. To assess household language, we use a measure that identifies those who spoke a language other than English in their household of origin while they were growing up. The analysis also examines how legal status shapes the likelihood of being insured. We identify those who reported being LPRs as well as those who are ‘likely undocumented.’ Those who are likely undocumented are 1.5-generation immigrants who arrived in the U.S. as minors and lack citizenship and LPR status.

Our fourth model adds measures for social contexts which may facilitate young adults' access to insurance—namely, postsecondary institutions and workplaces. For postsecondary schooling, we focus on college enrollment, since many in this population are not yet old enough to have completed their postsecondary education. In this survey, respondents were asked if they had enrolled in any educational programs since leaving high school. If they responded “yes,” they were asked to list all the different types of institutions they attended. We acknowledge that students’ postsecondary trajectories are quite complex and can include discontinuous enrollment in educational programs, switching schools, and simultaneous enrollment in more than one institution (Johnson and Muse, 2012). For parsimoniousness, we distinguish among those who: 1) never enrolled in college (but may have enrolled in a general educational development (GED), adult school, or vocational program); 2) enrolled in a community college (but never enrolled in a four-year college); and 3) enrolled in a four-year college or university. Individuals who completed their intended degrees are counted among those who attended a community college or a four-year university.

In examining whether or not respondents have any coverage, we distinguish among those not working (reference category), those who worked part-time at one or more jobs, and those with a single full-time job of 35 or more hours that might qualify them for employer-provided benefits. We exclude measures of employment status in models examining type of insurance because of collinearity concerns. (Non-working young adults cannot access employer-provided health insurance).

Our analyses include a fifth model that excludes non-Latino survey respondents. The purpose of this model is to identify predictors of insurance coverage among Latinos, and thus further unpack how family socioeconomic background, immigrant and cultural characteristics, and institutional ties shape coverage options.

We use sampling weights in our analyses. Missing data remained minimal, with less than 2% of observations missing for any of the above described variables. We assign non-missing values using multiple-imputation methods in STATA.

## 3 Results

### 3.1 Descriptive statistics

Table 1 shows descriptive statistics for the full sample and for Latino respondents alone. While 74% of young adults in California report having some type of health insurance, only 63% of Latinos do. In terms of type of insurance, similar percentages of the entire population and the Latino subsample have employer-provided coverage (19% and 21% respectively). However, while, 55% of all young adults report having insurance through some other source (including through their parents), only 42% of Latinos do.

Latinos represent the plurality of California's young adult population, comprising 44% of 18–26-year-olds who lived in the state as minors. Latino respondents come from disproportionately socioeconomically disadvantaged backgrounds. Only 15% of Latino young adults have a parent with a bachelor's degree, and 59% of Latino young adults grew up in low-income households. Notably, 78% of Latinos in the sample hail from immigrant families and most spoke a language other than English while growing up. The undocumented comprise a small percentage of the young adult population who obtained some portion of their K-12 education in the state.

Latino respondents are disproportionately represented among those who have not attended any college, and they are underrepresented among those who have attended a four-year institution. Although Latinos are slightly more likely to report full-time employment than the full sample, additional analyses not shown here indicate that this difference is not statistically significant.

### 3.2 Logistic regression results for having any health insurance

We present results for ethnoracial disparities and other determinants of having any insurance in Table 2. Model 1 addresses our first research question, demonstrating that overall, Latinos have much lower odds of being insured than young adults of all ethnoracial groups. Compared to Latinos, Whites exhibit nearly 3 times the odds of having any health insurance. Blacks, Asian-Pacific Islanders, and those who identify as Other race display 2 or more times the odds of insurance coverage than Latinos. These results are statistically significant and control for gender and age.

Model 2 includes indicators of socioeconomic status that play a role in ethnoracial differences in coverage. After adjusting for measures of family socioeconomic background (all statistically significant), Whites and those of Other race still exhibit statistically significant greater odds of health insurance coverage than Latinos.

Model 3 adds measures for immigrant family background, speaking another language at home, and legal documentation status. Accounting for these variables eliminates statistically significant ethnoracial inequalities in coverage. Interestingly, coming from an immigrant family has a positive, but non-significant association with having health insurance. At the same time, speaking a language other than English at home as a teenager—a rough indicator of ethnic cultural ties—significantly reduces the odds of having insurance (OR: 0.55, p < 0.01). In an alternative to Model 3 that excludes foreign language use in the home, findings reveal that coming from an immigrant family alone is not correlated with coverage (OR: 1.01, ns). This finding suggests that simply coming from an immigrant family neither improves nor reduces chances of obtaining health insurance, after accounting for socioeconomic background, legal status, and other variables. However, foreign language spoken at home remains statistically significant in an alternative model, also not shown here, that excludes immigrant family background. Thus, language spoken at home proves to be a stronger predictor of coverage than immigrant family background. Returning to Model 3 (as shown in Table 2), the results indicate that being an LPR does not correlate with the likelihood of having insurance coverage, yet being undocumented is associated with extremely low odds (0.36) of being insured (p < 0.01).

Non-family contexts also predict coverage, as demonstrated in Model 4. While results indicate that attending a community college is not associated with the likelihood of being insured, attending a four-year college is associated with 2.2 greater odds of being insured than those who have not attended any college (p < 0.001), after controlling for other variables. Results also demonstrate that having a full-time job is associated with 3.6 times the odds of coverage relative to not having a job at all (p < 0.001). This finding suggests that full-time jobs enable young adults to access employer-provided insurance or purchase their own.

Model 5 excludes non-Latinos from the analysis, thus examining factors that predict variations in insurance coverage among Latinos. Coming from a low-income family is strongly associated with being uninsured (p < 0.001). Tentative results indicate that Latinos from immigrant families are more likely to have insurance than those of native-born parentage (p < 0.10). Growing up speaking Spanish (or another language) at home corresponds with much lower odds of being insured (p < 0.001). As with the broader population, alternative analyses not presented here show that being from an immigrant family is not associated with coverage if speaking another language at home is excluded from Model 5. However, speaking another language at home remains negative and statistically significant if immigrant family background is excluded from the model.

The results in Model 5 also indicate that after controlling for other factors, Latinos who are employed full-time are more than 4 times more likely to have insurance than those who are not employed (p < 0.001). This finding underscores the importance of full-time employment in facilitating insurance access for this population. Interestingly, respondents’ own postsecondary educational enrollment is not a statistically significant predictor of coverage among Latino young adults.

### 3.3 Multinomial logistic regression results for having employer-provided or other health insurance

Addressing our third research question, Table 3 presents results from multinomial logistic regression analyses examining the likelihood of having either employer-provided insurance or coverage from some other source (including parents), relative to not having insurance at all. In Model 1, the results indicate that after controlling for gender and age, Whites exhibit 1.8 times greater odds of having employer-provided coverage when compared to Latinos (p < 0.01). The differences in employer-provided insurance between other ethnoracial groups and Latinos are not statistically significant. At the same time, statistically significant results for Model 1 demonstrate that Latinos are much less likely than all other ethnoracial groups to be covered by insurance from other sources.

When we add family socioeconomic measures in Model 2, the difference between Latinos and Whites in the likelihood of possessing employer-provided coverage disappears. However, Latinos remain less likely to possess other types of insurance when compared to other groups, except for Asian-Pacific Islanders.

In Model 3, we add immigration-related variables, and the results do not show statistically significant ethnoracial differences in coverage from employers or from other types of insurance, with one tentative exception. Those who identify as some other race exhibit more than twice the odds of having coverage when compared to Latinos (p < 0.10). Additionally, tentative evidence indicates that coming from an immigrant family is positively associated with having employer-provided coverage (p < 0.10). Meanwhile, foreign-language use in the home is negatively associated with possessing other types of coverage (OR 0.46, p < 0.001). LPR status is not statistically significant for either type of insurance. However, being undocumented is associated with a low likelihood of employer-provided coverage (OR: 0.46, ns) and coverage from another source (OR: 0.28, p < 0.01).

In Model 4, results indicate that community college enrollment is not a statistically significant predictor of neither having employer-provided or other sources of coverage. However, enrollment in a four-year college increases the likelihood of other coverage by 2.5 odds (p < 0.01), after accounting for other variables in the model.

Model 5 excludes non-Latinos from the analyses. Not surprisingly, Latinos from low-income backgrounds and the undocumented exhibit low odds of having employer-provided insurance. Meanwhile, those from immigrant families are tentatively 1.9 times as likely to obtain such insurance when compared to those with U.S.-born parents (p < 0.10), after controlling for other factors. At the same time, those from immigrant families are not at a disadvantage when it comes to possessing other types of insurance. Yet Latinos from low-income backgrounds, those who grew up speaking another language, and those who are undocumented all exhibit statistically significant lower odds of having other types of health insurance.

## 4 Discussion

Excluding first generation immigrants who arrived as adults, this study presents moderated estimates of the lack of insurance among Latino young adults. Nonetheless, the results align with prior research demonstrating that Latinos are much less likely to have health insurance than their peers of other ethnoracial backgrounds (Alegria et al., 2012, Ortega et al., 2015, Viruell-Fuentes et al., 2012). The findings presented here add to the literature by improving our understanding of how an immigrant family background, postsecondary school enrollment, and full-time employment attenuate the lower likelihood of having coverage. Importantly, this study also demonstrates that after accounting for family socioeconomic background, Latinos in this age group are as likely as their peers to have employer-provided insurance.

Prior assimilation research has suggested that coming from an immigrant family can provide a “second-generation advantage” in which the 1.5 and second generations demonstrate better outcomes than their native-born counterparts (Kasinitz et al., 2008). We find that this pattern may conditionally apply to insurance health coverage. Specifically, we offer tentative evidence that young adults from immigrant families *who speak English with their families* are more likely than similar peers from non-immigrant families to be insured by employers. Our findings point in the direction of what may be considered a “conditional second-generation advantage” because young adults who grew up speaking another language with their family are significantly less likely to have health insurance. In other words, when it comes to insurance coverage, speaking Spanish or another language at home suppresses any potential benefits of coming from an immigrant family. Being able to speak parents' native language is an indicator of ethnic cultural maintenance among the 1.5 and second generation (Portes and Rumbaut, 2001). It is possible that these young people who tend to be fluent in English (Alba et al., 2002, Rumbaut, 2004), but also speak Spanish or another foreign language, may engage in cultural practices that promote healthy behavior and perhaps decrease their perceived need for or use of health insurance, as found in other studies (Abraído-Lanza et al., 2005, Acevedo-Garcia and Bates, 2008, Acevedo-Garcia et al., 2010). Yet this study does not provide conclusive evidence that cultural practices or preferences predict insurance coverage. It is possible that young adults who grow up speaking their parents' language at home may encounter linguistic or other structural barriers to coverage not measured in this study. For example, they may disproportionately be the offspring of first generation immigrants who do not speak English. Their parents may lack access to jobs that offer health insurance because of language barriers (Wallace, 2015) and, as a result, these young adults may be less likely to obtain health insurance through their parents than similar peers who were raised in English-speaking households.

Notably, our empirical findings further confirm and shed light on the disadvantages experienced by undocumented immigrants (Castañeda and Melo, 2014, Joseph, 2016, Marrow and Joseph, 2015, Ortega et al., 2015, Zuckerman et al., 2011). The 1.5-generation immigrant young adults who lack legal documentation status have extremely little access to insurance coverage. This group suffered from little access to health insurance prior to the ACA (Bustamante et al., 2012, Bustamante et al., 2014). Because of legal documentation requirements, the ACA further reinforces the marginality of a group that already encounters a wide range of social and economic adversity (Terriquez, 2014; Gonzales, 2011).

This study also draws attention to how postsecondary institutions and employers shape coverage among Latinos. Our results remind readers that four-year colleges do not remain a key point of access for many young Latinos, in part because this ethnoracial group remains significantly underrepresented in four-year institutions. Instead, Latino young adults' labor market participation significantly improves their likelihood of coverage, a finding not shown in previous studies. Specifically, our results demonstrate that Latinos are as likely as their peers from similar family socioeconomic backgrounds to obtain employer-provided insurance. Accordingly, our study supports prior work indicating that Latino young adults raised in the U.S. tend to be better positioned in the labor market than first-generation immigrants (Waldinger et al., 2007) who have low rates of employer-provided coverage (Ortega et al., 2015). Still, Latino young adults remain disadvantaged when it comes to obtaining insurance from other sources, including through their parents.

### 4.1 Implications under the ACA and executive action on immigration

Latino young adults in California are likely to remain disproportionately uninsured as ACA implementation continues, but perhaps not to the same degree as shown in this article which relies on data collected in 2011. Latino young adults will probably continue to be less likely than other ethnoracial groups to receive insurance from government-provided sources—like Medicaid and Covered California, the state's insurance marketplace. Given anticipated ongoing barriers to coverage, it is projected that by 2019 Latinos will comprise 73% of the remaining uninsured in California, even though they will comprise approximately 41% of the state population. It is also estimated that young adults ages 19–29 year olds who comprise 18% of the state's population, will make up 28% of the remaining uninsured (Lucia et al., 2015).

Despite ACA limitations, coverage among Latinos increased between 2010 and 2014, particularly for those who were eligible for coverage through Medicaid and Covered California (Doty et al., 2015). It is possible that others will find increased access through employers, as the ACA requires large employers to provide coverage to their employees. And, while undocumented young adults will not directly benefit from the ACA, they will have increased access to insurance, as California policy now extends Medicaid coverage to undocumented youth who received a stay of deportation under President Obama's Deferred Action for Childhood Arrivals (DACA) program (Brindis et al., 2014). Moreover, efforts are underway to allow unauthorized immigrants to purchase coverage through the state's health exchange (Karlamangla, 2016). Thus, California is on track to increase access to coverage for undocumented Latinos.

Further investigation is needed to build on the findings presented here. Such research should address the limitations of this study, including its cross-sectional design; limited sample size; limited measures on types of insurance, and our inability to account for language barriers, parental employment, and parental documentation status. Future research should also account for the impact of different immigration policies that might shape access. For example, evidence indicates that, as of 2013, DACA increased coverage for some undocumented youth who received work permits through this executive action (Gonzales et al., 2014). Along with any initiatives to extend health insurance to the undocumented, any future policies that allow undocumented young adults or their parents to legally work and reside in the U.S. are likely to facilitate coverage (Bustamante et al., 2012, Bustamante et al., 2014, Ortega et al., 2015). However, without federal immigration reform that offers a reasonable pathway to citizenship for the undocumented, Latino young adults will remain vulnerable to being uninsured compared to those of other ethnoracial groups.

## Figures and Tables

**Table 1:** California young adult study (CYAS) 2011, sample description.

	Full Sample (N = 2200)	Latino Respondents (N = 1021)
*Health insurance coverage*		
Has health insurance	74%	63%
Employer provided health insurance	19%	21%
Health insurance from parents or other source	55%	42%
*Race/Ethnicity*		
Latino	44%	100%
White	35%	0%
Black	6%	0%
Asian-Pacific Islander	11%	0%
Other	4%	0%
*Gender*		
Male	52%	52%
Female	48%	48%
Average age	21.3	21.1
*Parental education*		
Parent(s) lack H.S. degree	16%	32%
Parent(s) have H.S. degree, no BA	49%	53%
Parent(s) with BA	35%	15%
Low-income background	38%	59%
Immigrant family	54%	78%
Speaks other language with family	45%	72%
*Citizenship/Legal status*		
U.S. citizen	92%	84%
Legal permanent resident	5%	9%
Undocumented	3%	7%
*Postsecondary enrollment*		
No college	35%	44%
Community college	32%	35%
Four-year college	33%	21%
*Employment status*		
Not employed	46%	46%
Part-time job(s)	30%	28%
Full-time job	24%	26%

Results may not add up to 100% because of rounding error.

**Table 2:** Odds ratios from logistic regressions of any health insurance, 2011 (N = 2200).

	Model 1	Model 2	Model 3	Model 4	Model 5^a^
*Race/ethnicity (ref. - Latino)*					
White	2.983***	1.535**	1.290	1.242	
	(0.437)	(0.255)	(0.246)	(0.242)	
Black	2.012**	1.416	1.224	1.541	
	(0.541)	(0.389)	(0.374)	(0.490)	
Asian-Pacific Islander	2.185**	1.252	1.140	1.083	
	(0.574)	(0.360)	(0.342)	(0.328)	
Other	4.462***	2.407*	1.980	2.380+	
	(1.863)	(1.034)	(0.868)	(1.103)	
Female	1.039	1.139	1.130	1.184	1.252
	(0.135)	(0.153)	(0.155)	(0.165)	(0.224)
Age	0.966	0.949*	0.948*	0.860***	0.891**
	(0.024)	(0.024)	(0.025)	(0.025)	(0.035)
*Parental education (ref.-parent with H.S. degree)*					
Parent(s) lack H.S.		0.592**	0.651*	0.626*	0.696+
		(0.110)	(0.123)	(0.120)	
Parent(s) with BA		1.528*	1.537*	1.311	0.835
		(0.281)	(0.280)	(0.236)	(0.234)
Low-income background		0.421***	0.451***	0.471***	0.360***
		(0.066)	(0.071)	(0.075)	(0.076)
Immigrant family			1.415	1.391	1.608+
			(0.311)	(0.304)	(0.434)
Speaks other language with family		0.546**	0.506***	0.370***	
			(0.110)	(0.103)	(0.104)
*Citizenship/legal status (ref.-citizen)*					
Legal permanent resident			1.087	1.154	0.979
			(0.378)	(0.398)	(0.390)
Undocumented			0.355**	0.385**	0.341**
			(0.129)	(0.144)	(0.132)
*Postsec. educ (ref.-no college)*					
Community college				1.087	0.969
				(0.178)	(0.203)
Four-year college				2.165***	1.344
				(0.410)	(0.341)
*Employment status (ref. not employed)*					
Part-time job(s)				1.160	1.313
				(0.182)	(0.288)
Full-time job				3.605***	4.320***
				(0.691)	(1.077)
Constant	3.454*	9.394***	11.479***	55.397***	38.266***
	(1.903)	(5.537)	(7.365)	(37.405)	(32.659)

Two-tailed tests: +p < 0.10, *p = <0.05, **p = <0.01, ***p = <0.001.

a Restricted to Latino sample only (n = 1021).

**Table 3:** Odds ratios from multinomial logistic regressions of employer and other health insurance, 2011 (N = 2200).

	Model 1		Model 2		Model 3		Model 4		Model 5^a^	
	Insurance from job v. no insurance	Other insurance v. no insurance	Insurance from job v. no insurance	Other insurance v. no insurance	Insurance from job v. no insurance	Other insurance v. no insurance	Insurance from job v. no insurance	Other insurance v. no insurance	Insurance from job v. no insurance	Other insurance v. no insurance
*Race/ethnicity (ref.Latino)*										
White	1.795**	3.670***	1.114	1.759***	1.160	1.353	1.140	1.265		
	(0.352)	(0.568)	(0.237)	(0.310)	(0.282)	(0.276)	(0.278)	(0.261)		
Black	0.670	2.755***	0.567	1.812*	0.607	1.436	0.617	1.497		
	(0.268)	(0.779)	(0.232)	(0.526)	(0.263)	(0.468)	(0.267)	(0.500)		
Asian-Pacific Islander	1.208	2.722***	0.821	1.472	0.722	1.368	0.675	1.123		
	(0.381)	(0.768)	(0.278)	(0.448)	(0.254)	(0.436)	(0.240)	(0.358)		
Other	1.779	6.143***	1.142	3.129*	1.087	2.421+	1.144	2.425+		
	(0.932)	(2.637)	(0.605)	(1.402)	(0.589)	(1.098)	(0.624)	(1.114)		
Female	0.747+	1.188	0.818	1.314+	0.825	1.310+	0.813	1.239	0.793	1.352
	(0.130)	(0.163)	(0.145)	(0.186)	(0.148)	(0.189)	(0.145)	(0.180)	(0.196)	(0.262)
Age	1.199***	0.881***	1.176***	0.862***	1.179***	0.859***	1.164***	0.820***	1.162***	0.861***
	(0.0393)	(0.0253)	(0.0388)	(0.0252)	(0.0392)	(0.0256)	(0.0384)	(0.025)	(0.052)	(0.037)
*Parental education (ref.-parent(s) with H.S. degree)*										
Parent(s) lack H.S.			0.955	0.451***	0.937	0.525**	0.936	0.524**	0.963	0.585*
			(0.251)	(0.089)	(0.245)	(0.107)	(0.245)	(0.109)	(0.283)	(0.138)
Parent(s) with BA			1.269	1.651**	1.258	1.680**	1.184	1.364+	0.811	0.854
			(0.281)	(0.319)	(0.279)	(0.322)	(0.265)	(0.255)	(0.284)	(0.265)
Low-income background			0.394***	0.440***	0.404***	0.485***	0.412***	0.527***	0.355***	0.403***
			(0.0820)	(0.0729)	(0.0850)	(0.0805)	(0.0876)	(0.0890)	(0.106)	(0.088)
Immigrant family					1.587+	1.348	1.556+	1.317	1.853+	1.603
					(0.429)	(0.315)	(0.416)	(0.307)	(0.687)	(0.462)
Speaks other language with family					0.718	0.485***	0.710	0.461***	0.670	0.315***
					(0.181)	(0.1052)	(0.177)	(0.1014)	(0.250)	(0.093)
*Citizenship/legal status (ref.-citizen)*										
Legal permanent resident					1.337	0.919	1.377	1.012	1.240	0.833
					(0.619)	(0.324)	(0.644)	(0.368)	(0.673)	(0.363)
Undocumented					0.461	0.280**	0.481	0.300**	0.421+	0.266**
					(0.234)	(0.120)	(0.243)	(0.129)	(0.216)	(0.117)
*Postsec. educ (ref.-no college)*										
Community college							1.071	1.101	1.017	0.977
							(0.230)	(0.192)	(0.283)	(0.219)
Four-year college							1.363	2.541***	1.009	1.467
							(0.334)	(0.511)	(0.341)	(0.401)
	0.0125***	14.270***	0.032***	43.726***	0.028***	62.850***	0.035***	136.821***	0.042**	78.741***
Constant	(0.009)	(8.678)	(0.025)	(28.606)	(0.023)	45.175	(0.028)	(98.521)	(0.045)	(73.226)

Two-tailed tests: +p = <0.10, *p = <0.05, **p = <0.01, ***p = <0.001.

a Restricted to Latino sample only (n = 1021).
